# The Influence of Tumor Burden Score and Lymph Node Metastasis on the Survival Benefit of Adjuvant Chemotherapy in Intrahepatic Cholangiocarcinoma

**DOI:** 10.1245/s10434-025-17013-5

**Published:** 2025-02-17

**Authors:** Jun Kawashima, Yutaka Endo, Selamawit Woldesenbet, Mujtaba Khalil, Miho Akabane, François Cauchy, Feng Shen, Shishir Maithel, Irinel Popescu, Minoru Kitago, Matthew J. Weiss, Guillaume Martel, Carlo Pulitano, Luca Aldrighetti, George Poultsides, Andrea Ruzzente, Todd W. Bauer, Ana Gleisner, Hugo Marques, Bas Groot Koerkamp, Itaru Endo, Timothy M. Pawlik

**Affiliations:** 1 Department of Surgery, The Ohio State University Wexner Medical Center and James Comprehensive Cancer Center, Columbus, OH USA; 2 Department of Gastroenterological Surgery, Yokohama City University School of Medicine, Yokohama, Japan; 3 Department of Transplant Surgery, University of Rochester Medical Center, Rochester, NY USA; 4 Department of Hepatobiliopancreatic Surgery, APHP, Beaujon Hospital, Clichy, France; 5 Department of Surgery, Eastern Hepatobiliary Surgery Hospital, Shanghai, China; 6 Department of Surgery, Emory University, Atlanta, GA USA; 7 Department of Surgery, Fundeni Clinical Institute, Bucharest, Romania; 8 Department of Surgery, Keio University, Tokyo, Japan; 9 Department of Surgery, Northwell Health, Manhasset, NY USA; 10 Department of Surgery, University of Ottawa, Ottawa, ON Canada; 11 Department of Surgery, Royal Prince Alfred Hospital, Camperdown, NSW Australia; 12 Department of Surgery, Ospedale San Raffaele, Milan, Italy; 13 Department of Surgery, Stanford University, Stanford, CA USA; 14 Department of Surgery, University of Verona, Verona, Italy; 15 Department of Surgery, University of Virginia, Charlottesville, VA USA; 16 Department of Surgery, University of Colorado Denver, Denver, CO USA; 17 Department of Surgery, Curry Cabral Hospital, Lisbon, Portugal; 18 Department of Surgery, Erasmus University Medical Centre, Rotterdam, The Netherlands

## Abstract

**Introduction:**

While postoperative adjuvant chemotherapy (AC) is generally recommended for intrahepatic cholangiocarcinoma (ICC), its benefit remains debated. This study aimed to identify patients that may benefit from AC following liver resection of ICC.

**Methods:**

Patients who underwent liver resection for ICC between 2000 and 2023 were identified from an international multi-institutional database. Individual multivariable Cox models were used to evaluate the interaction between each prognostic factor and the effect of AC on survival.

**Results:**

Among 1412 patients, 431 (30.5%) received AC. Both higher tumor burden score (TBS; hazard ratio [HR] 0.95, 95% confidence interval [CI] 0.91–1.00; *p* = 0.033) and metastatic lymph node status (HR 0.58, 95% CI 0.38–0.89; *p* = 0.014) demonstrated interactions with the survival benefit from receipt of AC. Interaction plots highlighted how AC was associated with improved survival beyond a TBS of approximately 6. Notably, among 555 (39.3%) patients with TBS <6 and N0 or Nx status, 5-year overall survival (OS) was no different between patients who received AC versus individuals who did not (55.1% [95% CI 48.9–62.1] vs. 58.7% [95% CI 49.8–69.2]; *p* = 0.900). In contrast, among 857 (60.7%) patients with TBS ≥6 or N1 status, AC was associated with improved 5-year OS (30.7% [95% CI 26.2–36.0] vs. 33.0% [95% CI 26.9–40.5]; *p* = 0.018).

**Conclusions:**

TBS and lymph node status may be useful in a multidisciplinary setting to inform decisions about AC planning for ICC patients following curative-intent resection.

**Supplementary Information:**

The online version contains supplementary material available at 10.1245/s10434-025-17013-5.

Intrahepatic cholangiocarcinoma (ICC) is the second most common primary hepatic malignancy, representing approximately 10–20% of all primary liver cancers.^1^ Over the last decade, the incidence of ICC has increased dramatically worldwide.^2,3^ Surgical resection is the only potentially curative treatment option; however, the prognosis remains poor.^4^ In particular, among patients with resectable tumors, survival typically ranges from 15 to 40 months.^4,5^ In contrast, patients with unresectable ICC have a much shorter survival, ranging from just 6–15 months.^4,6^ Given the poor outcomes following resection alone, the treatment strategy of combining surgery with adjuvant chemotherapy (AC) has been increasingly investigated.^7^

AC after curative-intent resection may minimize tumor recurrence and increase overall survival (OS) among patients with ICC, yet the universal use of AC remains controversial.^8^ Most data on the effects of AC on ICC have been extrapolated from clinical trials that included a heterogeneous group of patients with various biliary tract cancers.^7^ Of note, the results from these trials have been inconsistent.^9–12^ While the BILCAP and ASCOT trials demonstrated a survival benefit from AC, other trials such as BCAT and PRODIGE 12 failed to demonstrate a meaningful improvement in OS.^9–12^ Although these contrasting outcomes may be due to multiple factors, one possible explanation is that AC does not have a uniform effect across all ICC patients.^7^ Instead, specific subsets of patients may benefit from AC, while others do not.^7^ Identifying these subsets may improve clinical practice by facilitating a more personalized treatment approach.^7^ Of note, better stratification of patients would allow clinicians to avoid exposing patients who are unlikely to benefit from systemic chemotherapy to its toxic effects, while ensuring that individuals who are likely to benefit receive the needed treatment. Therefore, the objective of the current study was to characterize the impact of AC on the outcomes of patients with ICC following curative-intent resection. Utilizing a large, multi-institutional, international database, we sought to identify the subset of patients with ICC who benefited the most from AC.

## Methods

### Data Source and Patient Selection

Patients who underwent curative-intent liver resection, defined as surgery aiming for complete tumor removal with R0 margins, for ICC between 2000 and 2023 were identified from the International Intrahepatic Cholangiocarcinoma Study Group database that consisted of the 18 institutions (number, % of patients): Northwell Health, USA (21, 1.2%), Beaujon Hospital, France (76, 4.5%), Cleveland Clinic Foundation, USA (154, 9.1%), Curry Cabral Hospital, Portugal (85, 5.0%), Eastern Hepatobiliary Surgery Hospital, China (372, 21.9%), Emory University, USA (33, 1.9%), Erasmus University Medical Centre, The Netherlands (102, 6.0%), Fundeni Clinical Institute, Romania (188, 11.1%), Johns Hopkins University, USA (90, 5.3%), Keio University, Japan (36, 2.1%), University of Ottawa, Canada (56, 3.3%), The University of Sydney, Australia (69, 4.1%), Royal Prince Alfred Hospital, Australia (51, 3.0%), Stanford University, USA (76, 4.5%), University of Colorado Denver, USA (17, 1.0%), University of Virginia, USA (27, 1.6%), University of Verona, Italy (154, 9.1%), and Yokohama City University, Japan (89, 5.2%).^13^ Patients who had extrahepatic metastasis, diagnosed as M1 based on histopathological findings after curative-intent surgery, or an R2 resection margin were excluded. Additionally, individuals who had missing data on key clinicodemographic characteristics were not included. The Institutional Review Boards of each participating institution approved the study.

### Variables and Outcomes

Patient demographic and clinicopathologic variables included age, sex, American Society of Anesthesiologists (ASA) classification, year of surgery (i.e., 2000–2010, 2011–2023), cirrhosis, receipt of neoadjuvant chemotherapy (NAC), preoperative carbohydrate antigen 19-9 (CA19-9), type of surgery (i.e., major hepatectomy, minor hepatectomy), tumor size and number, tumor burden score (TBS), T category based on the American Joint Committee on Cancer (AJCC) 8th edition,^14^ nodal disease (i.e., N0: negative; N1: positive; Nx: not examined), margin status (i.e., R0, R1), microvascular invasion (MVI), morphological subtype (i.e., mass-forming [MF]; intraductal growth [IG]; periductal infiltrating [PI]; MF+PI), tumor grade (i.e., well, moderately, poorly differentiated, and undifferentiated), perineural invasion (PNI), and receipt of AC. Margin-free resection was defined as positive (<1 mm) or negative (≥1 mm).^7^ TBS, a composite index of tumor burden that incorporates maximum tumor size and number, was computed based on final pathology using the following formula: TBS^2^ = (maximum diameter)^2^ + (number of tumor)^2^.^15^ Liver resection was classified as major (≥3 segments) or minor (≤2 segments) according to ‘New World’ terminology.^16^ Receipt of AC was defined as the completion of at least one course of chemotherapy following surgery. Chemotherapy regimens were determined according to institutional protocols at each participating center.

### Statistical Analysis

Descriptive statistics were presented as median values (interquartile ranges [IQRs]) for continuous variables and frequencies (%) for categorical variables. Continuous variables were compared using the Mann–Whitney U or Kruskal–Wallis tests, as appropriate. Categorical variables were compared using the Chi-square or Fisher’s exact tests. Multiple imputations with chain equations (MICE) procedures were utilized to handle missing values.^17^ The primary outcome was OS, defined as the time interval between the dates of resection to the date of death from any cause or last follow-up. Patients alive at their last follow-up were censored at the time of their last known contact. Survival was estimated using the Kaplan–Meier method and log-rank tests.

A multivariable Cox proportional hazards model was constructed, including covariates selected based on clinical relevance and the previous literature. These covariates included age, sex, ASA physical status (PS), year of surgery, cirrhosis, receipt of NAC, CA19-9 level, type of surgery, TBS, T category, nodal status, margin status, MVI, morphological subtype, tumor grade, PNI, and receipt of AC.^5,7,13,18^ Hazard ratios (HRs) with 95% confidence intervals (CIs) were calculated to assess the impact of each prognostic factor on survival. Additionally, individual multivariable Cox models were utilized to evaluate the interaction between each prognostic factor and the effect of AC on survival separately.

Given the observed interaction between TBS and AC, adjusted survival curves were plotted to identify a threshold TBS value. This threshold was defined as the point below which AC was not associated with a significant survival difference and above which AC was associated with a survival benefit. Variables mentioned earlier were modeled using restricted cubic splines with three prespecified knots:^19^ the TBS values at which the survival curves for patients who did and did not receive AC began to diverge, as well as the values at which the CIs were narrowest.^19^ Based on a TBS threshold of 6, identified by restricted cubic splines analysis and the interaction observed between nodal status and AC, patients were grouped into an ‘effective group’ (TBS ≥6 and/or N1) and a ‘non-effective group’ (TBS <6 and N0/Nx). Kaplan–Meier survival curves and log-rank tests were used to evaluate the survival benefit of AC in each group. To further investigate the impact of AC on patients in the non-effective group, a multivariable Cox proportional hazards model was constructed for this subgroup to assess the association between AC and OS. Additionally, patients in the non-effective group were stratified into two subgroups (TBS 3–6 and TBS <3). Kaplan–Meier survival curves were used to compare OS in each subgroup examining who did versus did not receive AC.

To address potential baseline imbalances and validate the robustness of the findings, a sensitivity analysis was performed using a 1:1 propensity score matching (PSM) approach. A greedy nearest-neighbor algorithm without replacement was employed, using a caliper width of 0.1 standard deviations of the logit of the propensity score. Patients were matched based on age, sex, ASA PS, year of surgery, cirrhosis, receipt of NAC, CA19-9 level, type of surgery, TBS, T category, nodal status, margin status, MVI, morphological subtype, tumor grade, and PNI. Covariate balance was evaluated using standardized mean differences. Based on the findings from the primary analysis, patients in the PSM cohort were stratified into two groups to evaluate the survival benefit of AC more precisely: effective group, defined as patients with TBS ≥6 and/or N1 disease, and non-effective group, defined as patients with TBS <6 and N0/Nx disease. Kaplan–Meier survival curves and log-rank tests were used to evaluate the survival benefit of AC within each group. Statistical significance was set at α = 0.05. All analyses were performed using R version 4.2.2 (R Project for Statistical Computing, Vienna, Austria).

## Results

### Patient Demographics and Comparison of the Adjuvant Chemotherapy (AC) versus no AC Cohorts

Overall, 1696 patients underwent curative-intent surgery for ICC. Among the 1412 patients who met the inclusion criteria, 790 (55.9%) patients were male and median age was 61 years (IQR 52–70); 579 (41.0%) patients were ASA class >2 and 208 (14.7%) patients had cirrhosis. The median preoperative CA19-9 level was 41.0 IU/mL (IQR 13.0–192.0), and 852 (60.3%) patients underwent major hepatectomy; a small subset of patients received NAC (*n* = 99, 7.0%). On final pathology, 1207 patients (85.5%) had a solitary lesion with a median tumor size of 5.9 cm (IQR 4.0–8.0); median TBS was 6.1 (IQR 4.1–8.6). Approximately one-third of patients (*n* = 425, 30.1%) had T3 or T4 disease, and 286 patients (20.3%) had nodal metastasis (N1). A total of 446 (31.6%) patients had MVI with PI/MF+PI type, and poor or undifferentiated tumors; PNI was observed among 208 (14.7%), 257 (18.2%), and 309 (21.9%) patients, respectively. Of note, 252 (17.8%) patients had a positive resection margin (R1) (Table 1).

**Table 1:** Clinicopathological characteristics of the analytic cohort

Characteristics	All patients	Non-AC	AC	*p*-Value
	[*n* = 1412]	[*n* = 981 (69.5%)]	[*n* = 431 (30.5%)]	
Age, years (median [IQR])	61 [52–69]	62 [52–70]	61 [53–68]	0.396
Sex, male	790 (55.9)	565 (57.6)	225 (52.2)	0.069
ASA PS classification, >2	579 (41.0)	344 (35.1)	235 (54.5)	<0.001
Year of surgery				0.254
2000–2010	537 (38.0)	363 (37.0)	174 (40.4)	
2011–2023	875 (62.0)	618 (63.0)	257 (59.6)	
Cirrhosis	208 (14.7)	155 (15.8)	53 (12.3)	0.103
Neoadjuvant chemotherapy	99 (7.0)	48 (4.9)	51 (11.8)	<0.001
CA19-9				<0.001
<100 ng/mL	549 (38.9)	359 (36.6)	190 (44.1)	
≥100 ng/mL	287 (20.3)	160 (16.3)	127 (29.5)	
Unknown	576 (40.8)	462 (47.1)	114 (26.5)	
Major hepatectomy	852 (60.3)	515 (52.5)	337 (78.2)	<0.001
Multiple regions	205 (14.5)	131 (13.4)	74 (17.2)	0.073
Tumor size, cm (median [IQR])	5.9 [4.0–8.0]	5.8 [3.8–8.0]	6.0 [4.0–8.1]	0.469
TBS (median [IQR])	6.1 [4.1–8.6]	6.1 [4.0–8.5]	6.1 [4.2–8.6]	0.739
Pathological T stage				<0.001
T1/T2	987 (69.9)	723 (73.7)	264 (61.3)	
T3/T4	425 (30.1)	258 (26.3)	167 (38.7)	
Pathological N stage				<0.001
N0	484 (34.3)	317 (32.3)	167 (38.7)	
N1	286 (20.3)	147 (15.0)	139 (32.3)	
Nx	642 (45.5)	517 (52.7)	125 (29.0)	
Microvascular invasion	446 (31.6)	269 (27.4)	177 (41.1)	<0.001
Margin, positive	252 (17.8)	163 (16.6)	89 (20.6)	0.081
Morphologic type, PI/MF+PI	208 (14.7)	104 (10.6)	104 (24.1)	<0.001
Grade, poor/undifferentiated	257 (18.2)	150 (15.3)	107 (24.8)	<0.001
Perineural invasion	309 (21.9)	167 (17.0)	142 (32.9)	<0.001

Data are expressed as *n* (%) unless otherwise specified

*AC* Adjuvant chemotherapy, *ASA PS* American Society of Anesthesiologists physical status, *CA19-9* carbohydrate antigen, *TBS* Tumor burden score, *PI/MF+PI* Periductal infiltrating/mass-forming plus periductal infiltrating, *IQR* Interquartile range

Overall, 431 (30.5%) patients received AC. Patients who received AC were more likely to have had NAC (*n* = 51 [11.8%] vs. *n* = 48 [4.9%]; *p* < 0.001), high CA19-9 (CA19-9 ≥100 ng/mL: *n* = 127 [29.5%] vs. *n* = 160 [16.3%]; *p* < 0.001), and underwent major hepatectomy (*n* = 337 [78.2%] vs. *n* = 515 [52.5%]; *p* < 0.001). On pathology, patients with T3 or T4 disease (*n* = 167 [38.7%] vs. *n* = 258 [26.3%]; *p* < 0.001) and N1 disease (*n* = 139 [32.3%] vs. *n* = 147 [15.0%]; *p* < 0.001) were more likely to receive AC after hepatectomy. Moreover, patients who received AC were more likely to have MVI (*n* = 177 [41.4%] vs. *n* = 269 [27.4%]; *p* < 0.001), PI/MF+PI (*n* = 104 [24.1%] vs. *n* = 104 [10.6%]; *p* < 0.001), poorly or undifferentiated tumors (*n* = 107 [24.8%] vs. *n* = 150 [15.3%]; *p* < 0.001), and PNI (*n* = 142 [32.9%] vs. *n* = 167 [17.0%]; *p* < 0.001) (Table 1). No differences were observed among patients with versus without receipt of AC regarding age, sex, year of surgery, cirrhosis, TBS, or margin status (all *p* > 0.05).

### Survival and Risk Factors of Patients

After a median follow-up of 22.6 months (IQR 11.2–44.8), the unadjusted 5-year OS was 41.2% (95% CI 37.3–45.4) for patients who did not receive AC and 41.3% (95% CI 35.9–47.4) for patients who did receive AC. On multivariable Cox regression, ASA PS >2 (HR 1.39, 95% CI 1.17–1.65), cirrhosis (HR 1.46, 95% CI 1.19–1.80), CA19-9 ≥100 ng/mL (HR 1.41, 95% CI 1.14–1.74), TBS (HR 1.09, 95% CI 1.06–1.11), T3 or T4 diseases (HR 1.24, 95% CI 1.03–1.48), nodal disease (N1: HR 2.38, 95% CI 1.91–2.96; Nx: HR 1.41, 95% CI 1.16–1.72), and PI/MF+PI type (HR 1.25, 95% CI 1.01–1.55) were independent preoperative predictors of increased risk of death (Table 2). In contrast, receipt of AC was associated with a survival benefit in the overall cohort (HR 0.67, 95% CI 0.56–0.80).

**Table 2:** Cox regression of variables independently associated with survival

Characteristics	Univariate analysis			Multivariate analysis	
	HR (95% CI)	*p*-Value		HR (95% CI)	*p*-Value
Age	1.00 (1.00–1.01)	0.574		1.00 (0.99–1.01)	0.910
Sex					
Female	Ref			Ref	
Male	1.12 (0.96–1.30)	0.159		1.12 (0.95–1.31)	0.168
ASA PS classification					
≤2	Ref			Ref	
>2	1.19 (1.02–1.38)	0.026		1.39 (1.17–1.65)	<0.001
Year of surgery					
2000–2010	Ref			Ref	
2011–2023	0.90 (0.77–1.05)	0.177		0.92 (0.77–1.09)	0.313
Cirrhosis					
No	Ref			Ref	
Yes	1.41 (1.16–1.70)	<0.001		1.46 (1.19–1.80)	<0.001
Neoadjuvant chemotherapy					
No	Ref			Ref	
Yes	1.06 (0.78–1.45)	0.697		0.98 (0.71–1.36)	0.910
CA19-9					
<100 ng/mL	Ref			Ref	
≥100 ng/mL	1.65 (1.35–2.01)	<0.001		1.41 (1.14–1.74)	0.001
Unknown	1.25 (1.05–1.49)	0.011		1.20 (1.00–1.43)	0.053
Surgical procedure					
Minor hepatectomy	Ref			Ref	
Major hepatectomy	1.26 (1.07–1.47)	0.005		1.02 (0.84–1.23)	0.866
TBS	1.09 (1.07–1.11)	<0.001		1.09 (1.06–1.11)	<0.001
Pathological T stage					
T1/T2	Ref			Ref	
T3/T4	1.58 (1.34–1.85)	<0.001		1.24 (1.03–1.48)	0.022
Pathological N stage					
N0	Ref			Ref	
N1	2.42 (1.96–2.98)	<0.001		2.38 (1.91–2.96)	<0.001
Nx	1.27 (1.06–1.52)	0.009		1.41 (1.16–1.72)	0.001
Microvascular invasion					
No	Ref			Ref	
Yes	1.44 (1.23–1.69)	<0.001		1.02 (0.84–1.23)	0.866
Morphologic type					
MF, IG	Ref			Ref	
PI/MF+PI	1.42 (1.17–1.73)	<0.001		1.25 (1.01–1.55)	0.038
Grade					
Well/moderate	Ref			Ref	
Poor/undifferentiated	1.41 (1.17–1.69)	<0.001		1.16 (0.96–1.41)	0.133
Perineural invasion					
No	Ref			Ref	
Yes	1.42 (1.19–1.69)	<0.001		1.09 (0.88–1.34)	0.419
Margin					
R0	Ref			Ref	
R1	1.21 (1.00–1.46)	0.0048		1.06 (0.87–1.31)	0.546
Adjuvant chemotherapy					
No	Ref			Ref	
Yes	0.92 (0.78–1.09)	0.332		0.67 (0.56–0.80)	<0.001

*HR* Hazard ratio, *CI* Confidence interval, *ASA PS* American Society of Anesthesiologists physical status, *CA19-9* Carbohydrate antigen, *TBS* Tumor burden score, *MF* Mass-forming, *IG* Intraductal growth, *PI* Periductal infiltrating, *Ref* reference

### Interaction with Risk Factors and Survival Benefit of AC

**Fig. 1:**
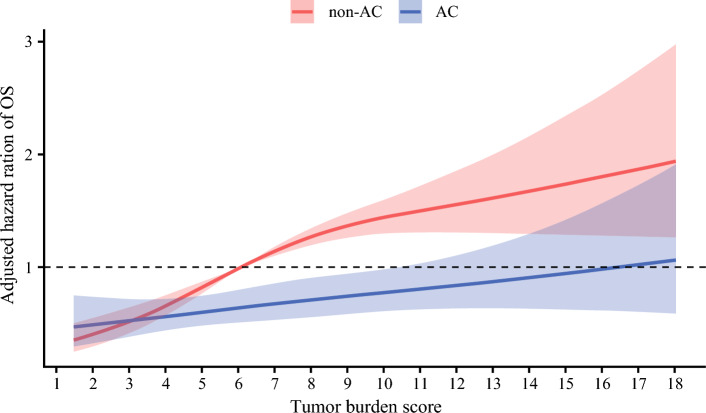
Plot of the interaction between tumor burden score, AC, and adjusted hazard of OS. *OS* overall survival, *AC* adjuvant chemotherapy

**Fig. 2:**
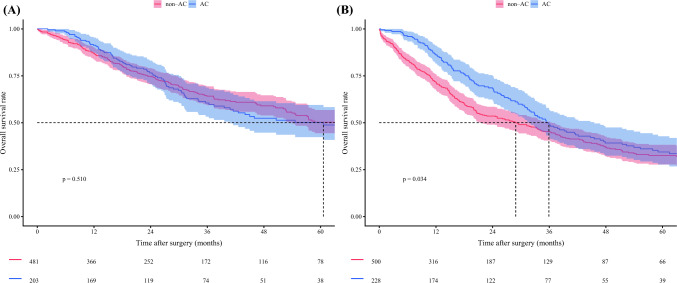
Kaplan–Meier estimates of 5-year overall survival curves. **A** Patients with TBS <6; **B** patients with TBS ≥6. *AC* adjuvant chemotherapy, *TBS* tumor burden score

**Fig. 3:**
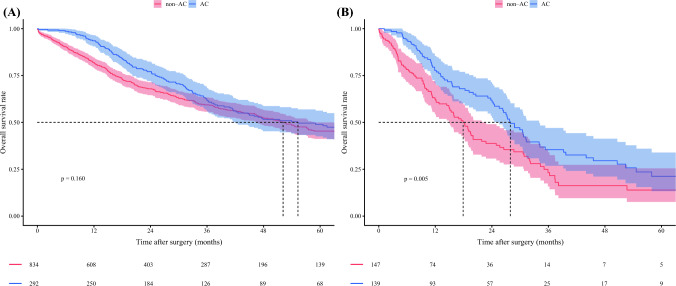
Kaplan–Meier estimates of 5-year overall survival curves. **A** Patients with N0 or Nx status; **B** patients with N1 status. *AC* adjuvant chemotherapy

**Fig. 4:**
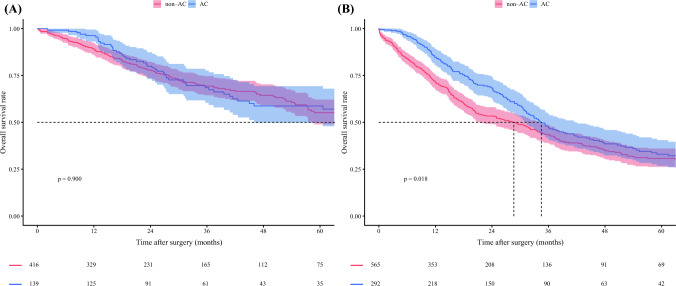
Kaplan–Meier estimates of 5-year overall survival curves. **A** ‘Non-effective’ group; **B** ‘effective’ group. *AC* adjuvant chemotherapy

Individual multivariable Cox models were developed to evaluate the interaction between each prognostic factor and the effect of AC on survival separately. Both TBS (HR 0.95, 95% CI 0.91–1.00; *p* = 0.033) and metastatic lymph node disease (HR 0.58, 95% CI 0.38–0.89; *p* = 0.014) demonstrated an interaction with receipt of AC, indicating that these factors influenced the relationship between AC and survival (Table 3). A plot of the interaction between TBS and receipt of AC demonstrated that the survival curves for patients receiving and not receiving AC diverged beyond a TBS of approximately 6. These data suggested that patients were less likely to experience a survival benefit from AC below a TBS threshold of 6, while being more likely to benefit as the TBS increased beyond 6 (Fig. 1). Notably, among patients with TBS <6, 5-year OS was no different based on receipt of AC (non-AC vs. AC: 50.2% [95% CI 44.4–56.7] vs. 50.1% [95% CI 42.2–59.4]; *p* = 0.510). In contrast, among patients with a TBS ≥6, AC was associated with improved 5-year OS (non-AC vs. AC: 32.6% [95% CI 27.8–38.2] vs. 34.5% [95% CI 27.8–42.8]; *p* = 0.034) (Fig. 2). Similarly, for patients with metastatic lymph nodes, AC was associated with improved 5-year OS (non-AC vs. AC: 13.9% [95% CI 7.6–25.4] vs. 21.2% [95% CI 13.2–33.9]; *p* = 0.005), while no survival benefit was observed in patients with N0 or Nx status (non-AC vs. AC: 45.3% [95% CI 41.1–49.9] vs. 48.9% [95% CI 42.5–56.2]; *p* = 0.160) (Fig. 3). Importantly, within the N0 or Nx group, patients with a TBS ≥6 experienced a survival benefit from AC (non-AC vs. AC: 35.7% [95% CI 30.4–42.0] vs. 41.2% [95% CI 33.1–51.4]; *p* = 0.026), while no such benefit was observed for individuals with a TBS <6 (55.1% [95% CI 48.9–62.1] vs. 58.7% [95% CI 49.8–69.2]; *p* = 0.900) (electronic supplementary material (ESM) Fig. 1). Based on these findings, patients with either a TBS ≥6 or metastatic lymph nodes were defined as the ‘effective group’ (*n* = 857, 60.7%), while the remaining patients were categorized as the ‘non-effective group’ (*n* = 555, 39.3%) (ESM Fig. 2). Of note, among patients in the non-effective group, 5-year OS was no different among patients who did versus did not receive AC (non-AC vs. AC: 55.1% [95% CI 48.9–62.1] vs. 58.7% [95% CI 49.8–69.2]; *p* = 0.900). In contrast, within the effective group, AC treatment was associated with improved 5-year OS (non-AC vs. AC: 30.7% [95% CI 26.2–36.0] vs. 33.0% [95% CI 26.9–40.5]; *p* = 0.018) [Fig. 4]. Notably, receipt of AC was not associated with improved OS among patients in the non-effective subgroup (HR 0.93, 95% CI 0.66–1.32) [ESM Table 1]. Additional analyses of patients with TBS 3–6 and TBS <3 demonstrated that there was no survival benefit of AC in either group. Specifically, among patients with TBS 3–6, 5-year OS was comparable between the non-AC and AC groups (54.5% [95% CI 47.6–62.4] vs. 58.3% [95% CI 48.1–70.6]; *p* = 0.800) (ESM Fig. 3).

**Table 3:** Summary of each interaction tested with adjuvant chemotherapy in a multivariable individual Cox proportional hazards model

Characteristics	HR (95% CI)	*p*-Value
Age	1.00 (0.99–1.02)	0.847
Sex, male	1.07 (0.77–1.50)	0.673
ASA PS classification, >2	0.87 (0.61–1.25)	0.455
Year of surgery, 2011–2023	1.25 (0.89–1.74)	0.197
Cirrhosis	0.85 (0.55–1.30)	0.445
Neoadjuvant chemotherapy	1.01 (0.53–1.90)	0.984
CA19-9, ≥100 ng/mL	1.03 (0.68–1.56)	0.895
CA19-9, unknown	0.95 (0.64–1.41)	0.802
Major hepatectomy	0.92 (0.63–1.34)	0.658
TBS	0.95 (0.91–1.00)	0.033
pT3/4	0.81 (0.58–1.14)	0.227
pN1	0.58 (0.38–0.89)	0.014
pNx	0.67 (0.44–1.01)	0.053
Microvascular invasion	0.88 (0.61–1.25)	0.465
Morphologic type, PI/MF+PI	0.66 (0.43–1.00)	0.051
Grade, poor/undifferentiated	1.31 (0.89–1.94)	0.175
Perineural invasion	0.93 (0.63–1.36)	0.694
Margin, positive	0.97 (0.63–1.49)	0.874

*HR* Hazard ratio, *CI* Confidence interval, *ASA PS* American Society of Anesthesiologists physical status, *CA19-9* Carbohydrate antigen 19-9, *TBS* Tumor burden score, *PI/MF+PI* Periductal infiltrating/mass-forming plus periductal infiltrating

### Additional Analysis Using Propensity Score Matching

PSM identified 392 pairs of patients with balanced background characteristics (ESM Table 2). The unadjusted 5-year OS was 35.6% (95% CI 30.2–42.0) for patients who did not receive AC and 42.7% (95% CI 37.1–49.2) for individuals who did. After matching, the interaction plot revealed that the survival curves of patients who received AC versus patients who did not began to diverge beyond a TBS of 6 (ESM Fig. 4). In a subsequent sensitivity analysis, the cohort was divided into effective and non-effective groups. Among the 260 patients (33.2%) in the non-effective group, AC did not improve survival compared with no AC (non-AC vs. AC: 58.1% [95% CI 49.1–70.5] vs. 59.2% [95% CI 50.1–70.0]; *p* = 0.870). Of note, among 524 patients (66.8%) in the effective group, AC was associated with improved survival versus no AC (non-AC vs. AC: 25.1% [95% CI 19.4–32.4] vs. 34.3% [95% CI 27.8–42.3]; *p* < 0.001) (ESM Fig. 5).

## Discussion

For patients with localized ICC, the standard of care involves surgical resection with negative margins and concomitant lymphadenectomy.^20–22^ Despite these measures, recurrence remains common, underscoring the importance of a multidisciplinary approach to manage this aggressive cancer.^23^ Although the utilization of AC for resected ICC has increased as part of this multidisciplinary approach, there have been conflicting results regarding the efficacy of AC among patients with ICC following curative-intent resection.^9–12,24,25^ A possible reason for these inconsistent outcomes is the lack of clear criteria for selecting patients who would benefit most from AC.^7,26,27^ As such, the current study was important because we identified a notable relationship between a higher TBS and metastatic lymph node status with the survival benefit of AC among patients undergoing curative-intent resection for ICC. Furthermore, using restricted cubic spline analysis, AC was demonstrated not to be associated with a survival benefit among patients with a TBS <6, yet was associated with improved survival with TBS ≥6. Subsequently, patients with TBS ≥6 or metastatic lymph nodes were identified as candidates for more ‘effective’ AC. To this point, patients in the ‘effective’ group had improved survival with receipt of AC (non-AC vs. AC: 30.7% [95% CI 26.2–36.0] vs. 33.0% [95% CI 26.9–40.5]; *p* = 0.018). In contrast, no survival benefit of AC was observed in the ‘non-effective’ group (no AC vs. AC: 55.1% [95% CI 48.9–62.1] vs. 58.7% [95% CI 49.8–69.2]; *p* = 0.900). Of note, the benefit of AC among patients with TBS ≥6 and/or N1 disease remained even after balancing other clinicopathological characteristics among non-AC versus AC patients using PSM.

The role of AC among patients with resectable ICC remains debatable.^7^ In the BILCAP trial, the primary endpoint of OS was not met in the intention-to-treat analysis, as OS was 51.1 months in the capecitabine group versus 36.4 months in the observation group (HR 0.81, 95% CI 0.63–1.04; *p* = 0.097); however, the prespecified per-protocol analysis suggested that capecitabine improved OS (HR 0.75, 95% CI 0.58–0.97; *p* = 0.028).^9^ In contrast, the PRODIGE trial demonstrated no survival benefit with adjuvant gemcitabine and oxaliplatin (GEMOX), even though the safety and tolerance profile were acceptable.^12^ The discrepancy in results between these two randomized clinical trials has been attributed to differences in patients’ charateristics.^28^ For instance, BILCAP had more patients with R1 (38% vs.13%) and N1 (47% vs. 38%) disease compared with PRODIGE, suggesting that AC may be more beneficial for patients with adverse prognostic factors than individuals with favorable clinicopathologic characteristics.^9,12^ In fact, the BILCAP trial demonstrated a statistically significant difference in OS after adjusting for nodal status, indicating that node-positive patients might derive greater benefit from AC.^9^ Likewise, in a subgroup analysis of the ASCOT trial, survival benefit of AC was observed in node-positive patients, whereas there was no survival benefit in node-negative patients.^10^ Consistent with these findings, the current study demonstrated that AC was associated with increased survival among patients with N1 disease (5-year OS, non-AC vs. AC: 13.9% [95% CI 7.6–25.4] vs. 21.2% [95% CI 13.2–33.9]; *p* = 0.005). There was however no impact of AC on survival among patients with N0 or Nx status (non-AC vs. AC: 45.3% [95% CI 41.1–49.9] vs. 48.9% [95% CI 42.5–56.2]; *p* = 0.160).

TBS, originally proposed by Sasaki and colleagues, is a novel ‘metro-ticket’ tool based on tumor size and number, initially developed to predict prognosis following colorectal liver metastasis (CRLM).^15,29,30^ This easy-to-use and accurate prognostic tool has also been validated for other liver cancers such as hepatocellular carcinoma and ICC, with its utility demonstrated in several studies.^31–34^ For instance, both OS and recurrence-free survival have been noted to worsen incrementally with higher TBS following liver resection for ICC, with a 5-year OS of only 17.3% among patients with high TBS.^33^ A separate study noted that ICC patients following liver resection with a higher TBS were more likely to experience early recurrence.^34^ Similar to these findings, the current study also noted that a higher TBS was associated with worse long-term outcomes, reinforcing the notion that TBS is an indicator of oncologic burden among ICC patients after liver resection. However, there has been limited research on the utility of AC in relation to TBS.^7,33^ In particular, whether TBS can be used to stratify patients based on their likelihood of benefiting from AC remains unclear. Importantly, the current study demonstrated that TBS impacted the survival benefit of AC among ICC patients. Furthermore, by employing restricted cubic splines, we identified a critical TBS threshold of 6 beyond which AC was associated with a survival benefit. These findings were particularly noteworthy because even in the setting of no nodal disease, patients with a TBS of ≥6 had an improved survival with AC administration. This finding challenges the conventional notion that the absence of nodal disease is an indicator that AC will have limited benefit. Rather, the data suggest that incorporating TBS into clinical decision making could refine patient selection for AC, particularly in the setting of node-negative disease.

Chemotherapy-related adverse events (AEs) can significantly impact patient quality of life, leading to increased healthcare utilization, higher costs, and potential delays or discontinuation of treatment.^35,36^ Notably, the BILCAP study revealed that 44% of patients receiving capecitabine experienced at least one grade 3 toxicity.^9^ The high incidence of severe AEs underscores the need to carefully balance the potential survival benefits of AC with the substantial risks of toxicity.^37^ In the current study, to optimize therapeutic decision making, patients who underwent liver resection for ICC were stratified into two distinct groups based on their likelihood of deriving a benefit from AC. The ‘effective’ group was characterized by a TBS of ≥6 or the presence of metastatic nodal disease, while the ‘non-effective’ group included patients with a lower TBS and no nodal involvement. The findings demonstrated a survival benefit from AC for a specific subset of patients in which the intensity of treatment may be justified by the greater likelihood of improved long-term outcomes. In contrast, no such benefit was observed in the non-effective group, suggesting that these patients may be spared the potential harms of AC without compromising their prognosis. However, caution is warranted when interpreting the TBS cut-off and stratification into the effective and non-effective groups. Among patients with a TBS ≥6 within the effective group, there was a clear separation in survival up to 24 months, but this difference began to converge after 24–36 months. One plausible explanation was the early benefit of AC in delaying recurrence, which diminished over time.^34,38,39^ Additionally, the relatively short median follow-up time of 22.6 months (IQR 11.2–44.8) likely contributed to a limited number of long-term events, reducing the statistical power to detect differences beyond 36 months. Furthermore, the limited sample size may have also contributed to a potential type II error, particularly among patients in the non-effective group. Future studies with larger cohorts and extended follow-up periods are needed to address these limitations and provide a more comprehensive understanding of the long-term impact of AC.

Several limitations should be considered when interpreting the results of the current study. Due to the rarity of ICC, a large international multi-institutional database containing patients from multiple decades was necessary to obtain a sufficient sample size. While the multicenter aspect was a strength, variations in treatment approaches and types of AC among institutions and over time may have influenced the results. The retrospective nature of this study also introduced potential selection bias and unmeasured confounding factors that may have affected patient outcomes. In the current study, CA19-9 data were analyzed as a categorical variable (≥100, <100, or unknown). The categorization of these data in this manner may have limited the ability to evaluate AFP as a predictor of AC benefit. Additionally, the analysis included various chemotherapy regimens, therefore it was not possible to assess the type of chemotherapy or to determine the best regimen for ICC. In particular, detailed data regarding the total duration of chemotherapy or the proportion of patients who discontinued treatment prematurely were not available. The threshold of TBS 6 was identified through restricted cubic spline analysis as a point in which the survival benefit of AC began to diverge. While this cut-off provided a practical framework for patient stratification, validating its generalizability to other populations will be needed. Additionally, there may have been an increased risk of type I error due to the evaluation of multiple interaction terms across various models. Although these interaction terms were chosen based on clinical relevance and previous research, the possibility of false-positive findings cannot be completely ruled out. To mitigate these concerns, future studies using independent datasets and external validation will be needed to confirm the robustness of these findings, assess their generalizability, and refine their clinical applicability.

## Conclusion

AC was not associated with a survival benefit among patients with a TBS below 6 and node-negative disease. In contrast, patients with a TBS of ≥6 and/or metastatic lymph nodes did experience a survival benefit from AC administration following curative-intent hepatic resection of ICC. These findings suggest that TBS and lymph node status may be useful in the multidisciplinary setting to inform decisions about adjuvant therapy planning for ICC patients following curative-intent resection.

## Supplementary Information

Below is the link to the electronic supplementary material.

Supplementary file1 (DOCX 23 kb)

Supplementary file2 (DOCX 724 kb)
