# Supplementary material for: The Influence of Tumor Burden Score and Lymph Node Metastasis on the Survival Benefit of Adjuvant Chemotherapy in Intrahepatic Cholangiocarcinoma
Source: Ann Surg Oncol. 2025 Feb 17;32(6):4341–51. doi: 10.1245/s10434-025-17013-5 (PMC12049291; doi:10.1245/s10434-025-17013-5)
Supplement: Supplementary file 1 — Supplementary file1 (DOCX 23 kb) [file 10434_2025_17013_MOESM1_ESM.docx]

Supplementary Table 1. Multivariable Cox regression of variables independently associated with survival in the non-effective group.

|  | Multivariate analysis | |
| --- | --- | --- |
| Characteristics | HR 95%CI | *P* value |
| Age | 1.01 [0.99, 1.02] | 0.415 |
| Sex |  |  |
| Female | Ref |  |
| Male | 1.18 [0.85, 1.63] | 0.325 |
| ASA PS classification |  |  |
| classification, ≤ 2 | Ref |  |
| classification, > 2 | 1.17 [0.84, 1.64] | 0.350 |
| Year of surgery |  |  |
| 2000-2010 | Ref |  |
| 2011-2023 | 0.95 [0.69, 1.31] | 0.750 |
| Cirrhosis |  |  |
| No | Ref |  |
| Yes | 1.47 [1.01, 2.14] | 0.046 |
| Neoadjuvant chemotherapy |  |  |
| No | Ref |  |
| Yes | 1.32 [0.65, 2.65] | 0.442 |
| CA19-9 |  |  |
| < 100 ng/mL | Ref |  |
| ≥ 100 ng/mL | 1.19 [0.75, 1.91] | 0.460 |
| Unknown | 1.13 [0.82, 1.56] | 0.439 |
| Surgical procedure |  |  |
| Minor hepatectomy | Ref |  |
| Major hepatectomy | 0.74 [0.53, 1.05] | 0.095 |
| TBS | 1.16 [1.02, 1.32] | 0.021 |
| Pathological T stage |  |  |
| T1/T2 | Ref |  |
| T3/T4 | 1.14 [0.77, 1.69] | 0.515 |
| Microvascular invasion |  |  |
| No | Ref |  |
| Yes | 1.11 [0.74, 1.66] | 0.607 |
| Morphologic type, |  |  |
| MF, IG | Ref |  |
| PI/MF+PI | 1.90 [1.22, 2.97] | 0.005 |
| Grade |  |  |
| Well/moderate | Ref |  |
| Poor/undifferentiated | 1.57 [1.00, 2.47] | 0.050 |
| Perineural invasion |  |  |
| No | Ref |  |
| Yes | 1.25 [0.83, 1.90] | 0.290 |
| Margin |  |  |
| R0 | Ref |  |
| R1 | 1.28 [0.88, 1.86] | 0.193 |
| Adjuvant chemotherapy |  |  |
| No | Ref |  |
| Yes | 0.93 [0.66, 1.32] | 0.701 |

**^ASA PS^**^, American Society of Anesthesiologists Physical Status;^ **^CA19-9^**^, Carbohydrate antigen;^ **^TBS^**^, Tumor burden score;^ **^MF,^** ^mass-forming;^ **^IG,^** ^intraductal growth;^ **^PI^**^,^ ^periductal infiltrating^

Supplementary Table 2. Background characteristics of propensity score-matched study patients stratified receipt of adjuvant chemotherapy.

| Characteristics | Non-AC | AC | SMD |
| --- | --- | --- | --- |
|  | n = 392 | n = 392 |  |
| Age, y, median (IQR) | 62 [50, 70] | 61 [53, 69] | 0.021 |
| Sex, male, n (%) | 201 (51.3) | 207 (52.8) | 0.031 |
| ASA- PS classification, > 2, n (%) | 211 (53.8) | 201 (51.3) | 0.051 |
| Year of surgery, 2011-2023, n (%) | 232 (59.2) | 243 (62.0) | 0.057 |
| Cirrhosis, n (%) | 52 (13.3) | 45 (11.5) | 0.054 |
| Neoadjuvant chemotherapy, n (%) | 36 (9.2) | 43 (11.0) | 0.059 |
| CA19-9, n (%) |  |  | 0.049 |
| < 100 ng/mL | 187 (47.7) | 178 (45.4) |  |
| ≥ 100 ng/mL | 99 (25.3) | 106 (27.0) |  |
| Unknown | 106 (27.0) | 108 (27.6) |  |
| Major hepatectomy, n (%) | 300 (76.5) | 299 (76.3) | 0.018 |
| Multiple regions, n (%) | 72 (18.4) | 67 (17.1) | 0.033 |
| Tumor size, cm, median (IQR) | 6.0 [4.0, 8.0] | 6.0 [4.0, 8.2] | 0.006 |
| TBS, median (IQR) | 6.1 [4.1, 8.8] | 6.1 [4.2, 8.8] | 0.014 |
| Pathological T3/T4 stage, n (%) | 145 (37.0) | 149 (38.0) | 0.021 |
| Pathological N stage, n (%) |  |  | 0.084 |
| N0 | 164 (41.8) | 149 (38.0) |  |
| N1 | 108 (27.6) | 120 (30.6) |  |
| Nx | 120 (30.6) | 123 (31.4) |  |
| Microvascular invasion, n (%) | 157 (40.1) | 162 (41.3) | 0.026 |
| Morphologic type, PI/MF+PI, n (%) | 77 (19.6) | 87 (22.2) | 0.063 |
| Grade, poor/undifferentiated, n (%) | 96 (24.5) | 93 (23.7) | 0.018 |
| Perineural invasion, n (%) | 123 (31.4) | 130 (33.2) | 0.038 |
| Margin, positive, n (%) | 80 (20.4) | 81 (20.7) | 0.006 |

**^AC^**^, adjuvant chemotherapy;^ **^ASA PS^**^, American Society of Anesthesiologists Physical Status;^ **^CA19-9^**^, Carbohydrate antigen;^ **^TBS^**^, Tumor burden score;^ **^PI/MF+PI^**^,^ ^periductal infiltrating/ mass forming plus periductal infiltrating^
