# Supplementary material for: The Influence of Tumor Burden Score and Lymph Node Metastasis on the Survival Benefit of Adjuvant Chemotherapy in Intrahepatic Cholangiocarcinoma
Source: Ann Surg Oncol. 2025 Feb 17;32(6):4341–51. doi: 10.1245/s10434-025-17013-5 (PMC12049291; doi:10.1245/s10434-025-17013-5)

Supplementary Figure 1: Kaplan-Meier estimates of 5-year overall survival curves. (A) patients with tumor burden score (TBS) < 6 and N0 / Nx status, (B) patients with TBS ≥ 6 and N0 / Nx status


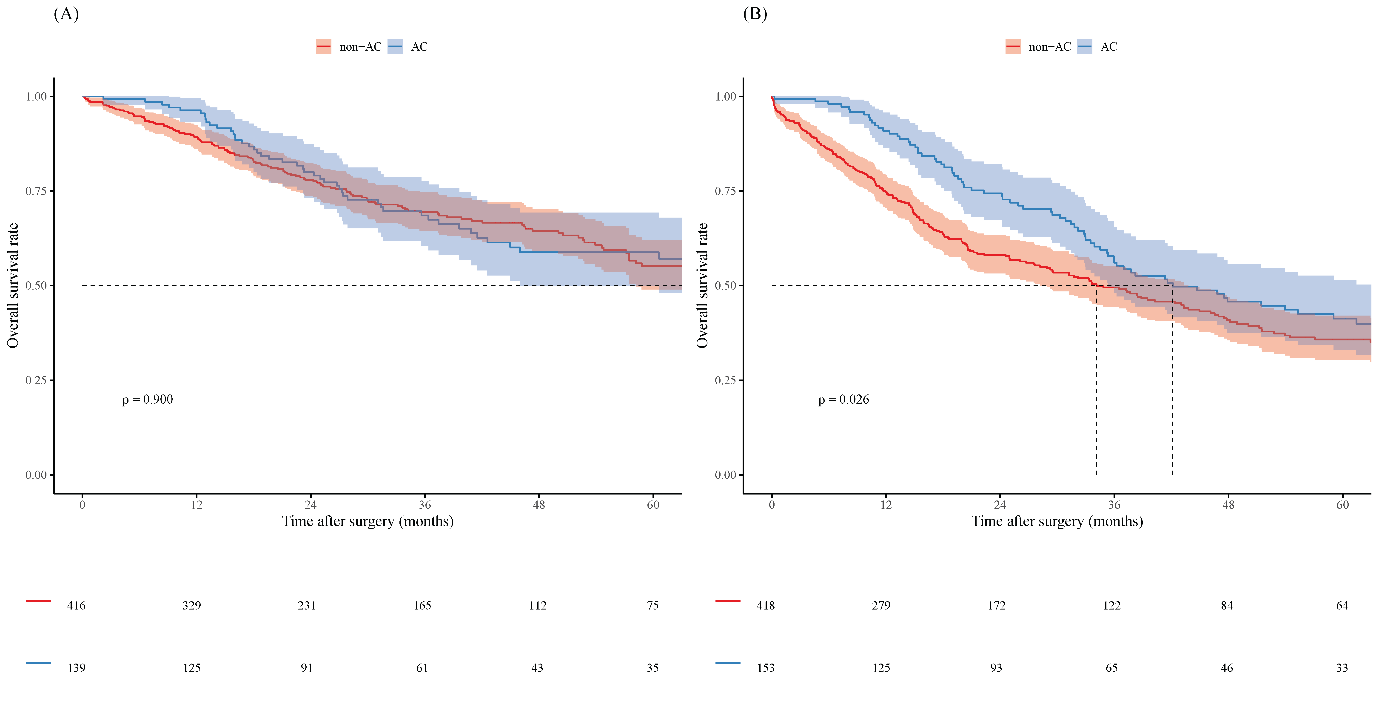


Supplementary Figure 2: Schematic presentation of “effective” group and “non-effective” group


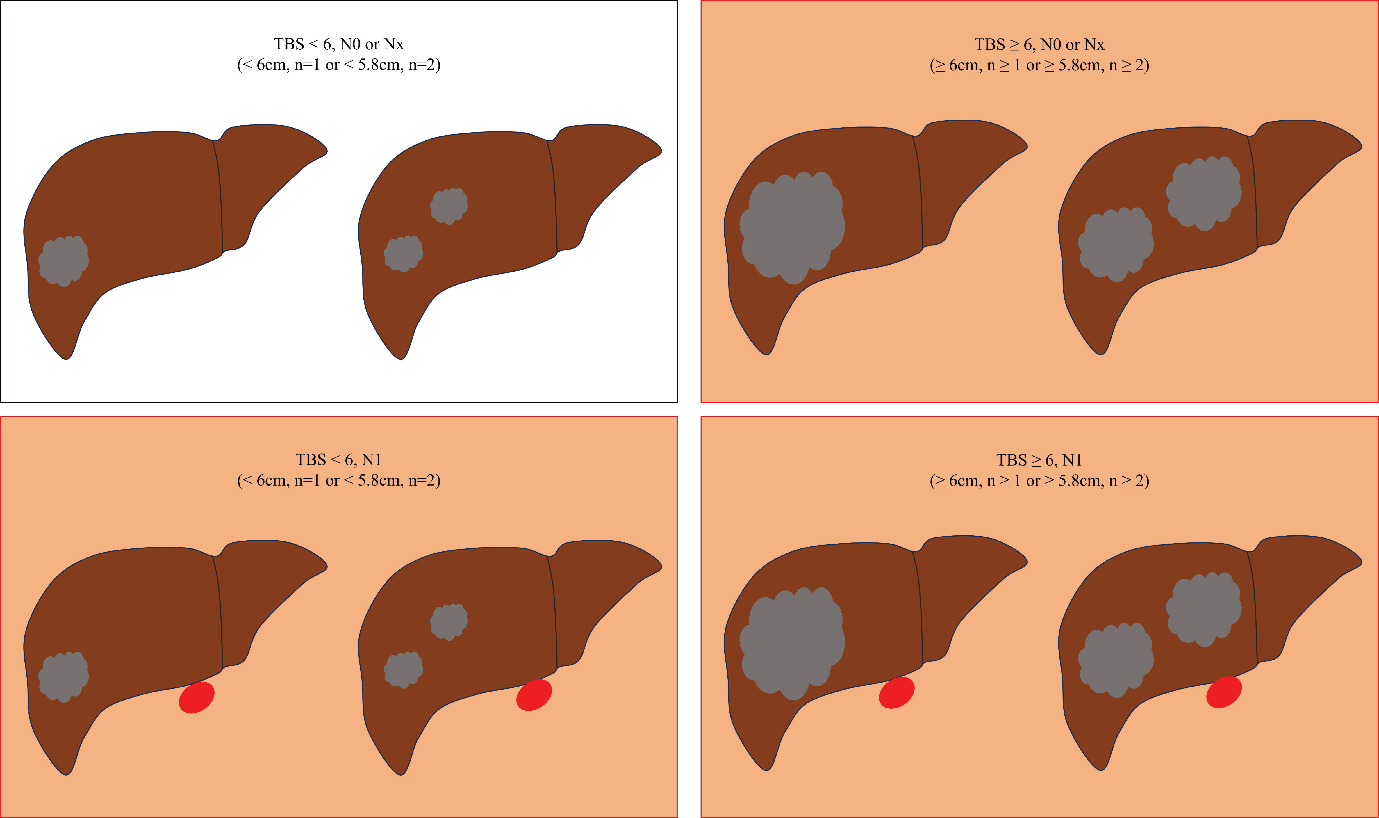


Supplementary Figure 3: Kaplan-Meier estimates of 5-year overall survival (OS) curves stratified by tumor burden score (TBS): (A) TBS < 3 and (B) TBS 3-6, in the “non-effective” group


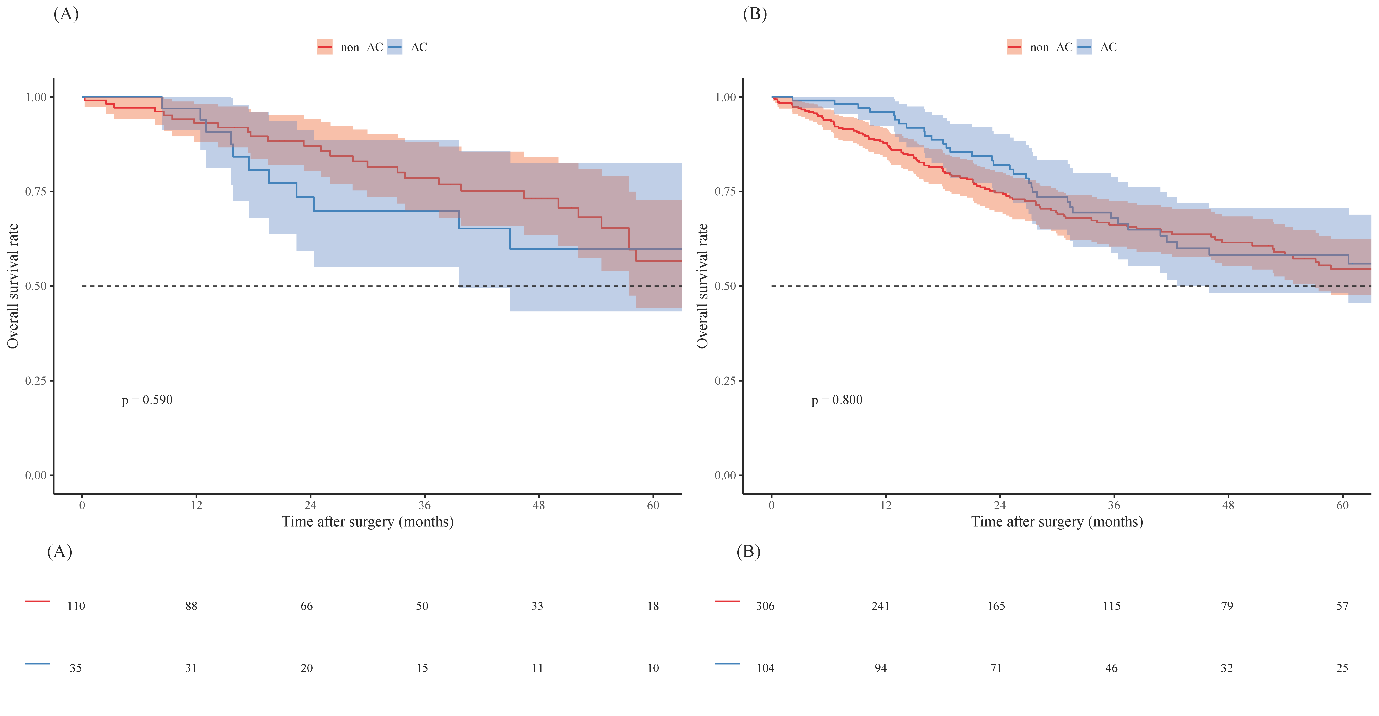


Supplementary Figure 4: A plot of the interaction between tumor burden score (TBS), adjuvant chemotherapy (AC), and adjusted hazard of overall survival (OS) after propensity score matching


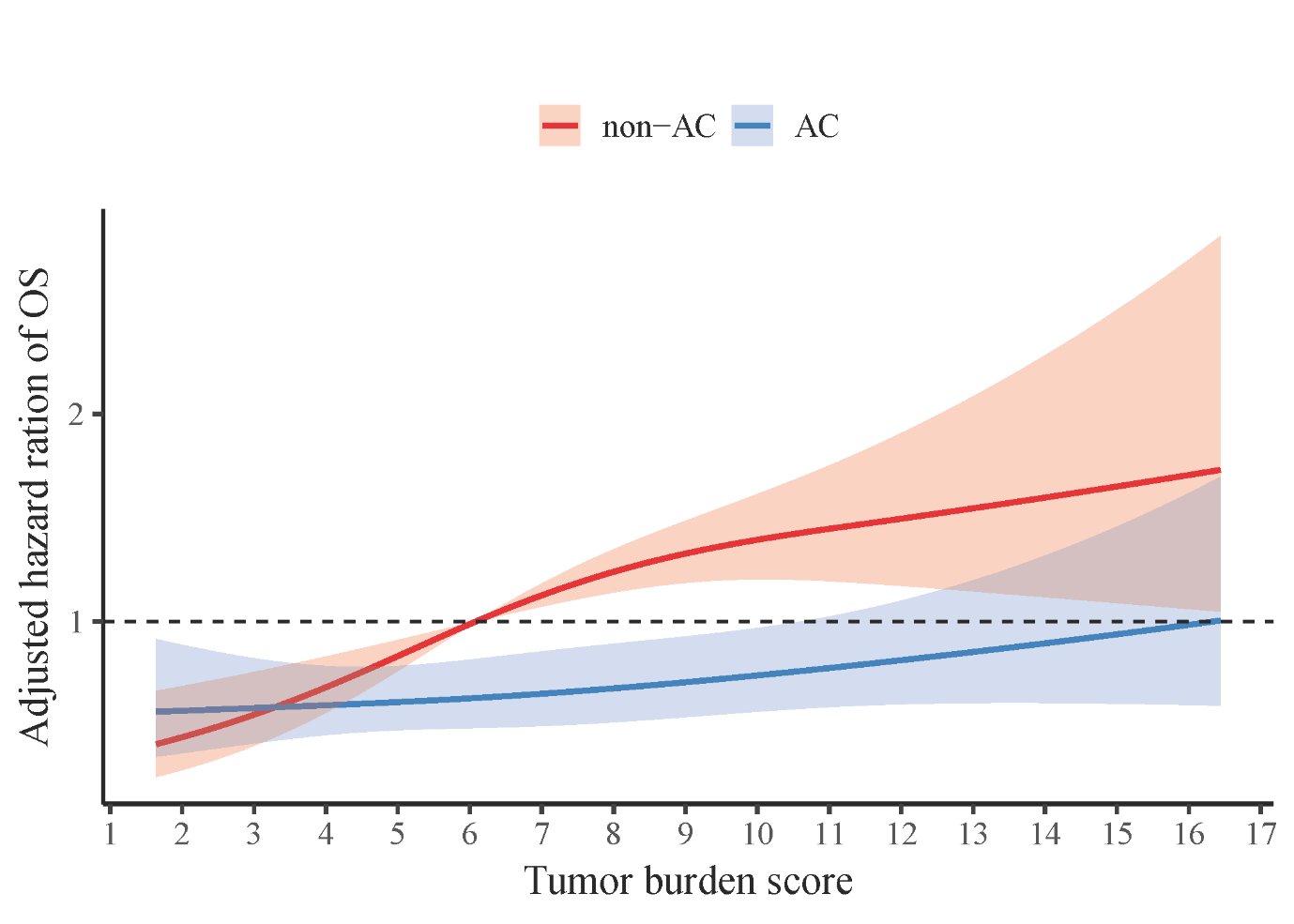


Supplementary Figure 5: Kaplan-Meier estimates of 5-year overall survival curves after propensity score matching. (A) “non-effective” group, (B) “effective” group


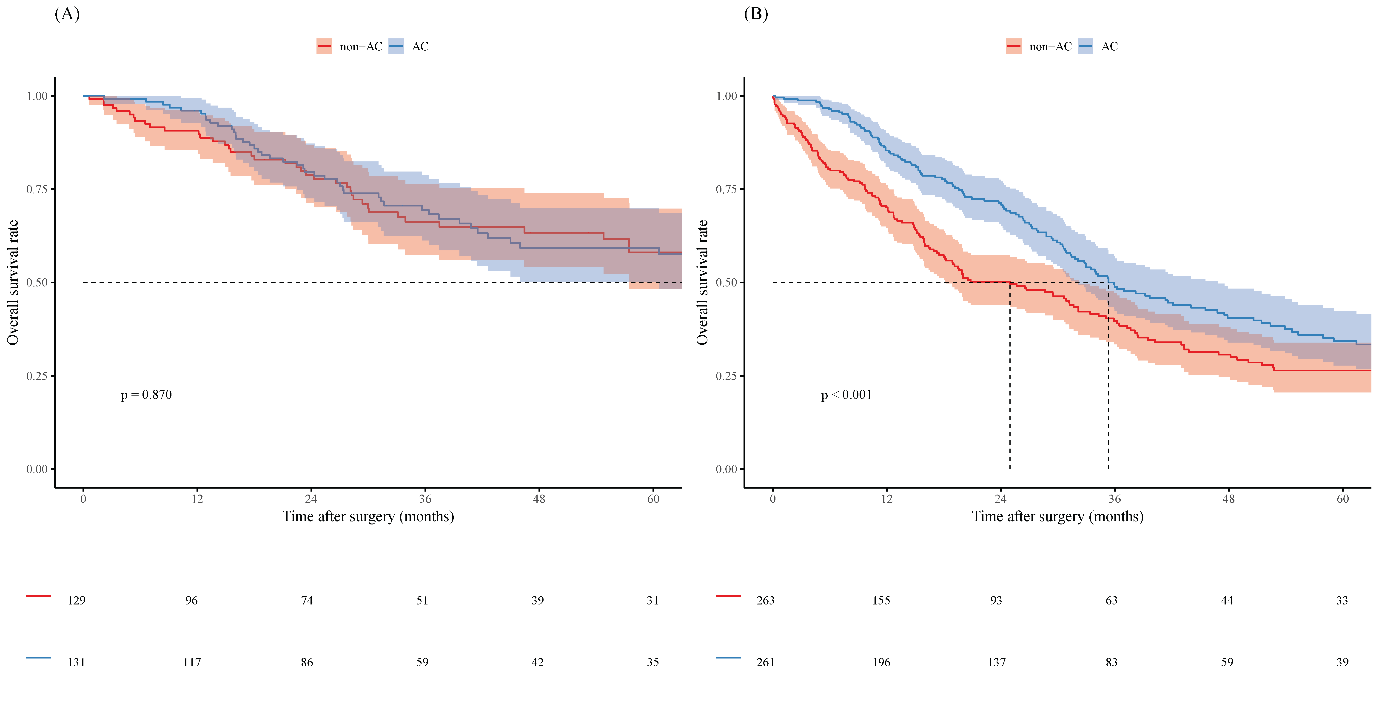

Supplement: Supplementary file 2 — Supplementary file2 (DOCX 724 kb) [file 10434_2025_17013_MOESM2_ESM.docx]
